# Parsimonious Determination of the Optimal Infectious Dose of a Pathogen for Nonhuman Primate Models

**DOI:** 10.1371/journal.ppat.1005100

**Published:** 2015-08-18

**Authors:** Mario Roederer

**Affiliations:** ImmunoTechnology Section, Vaccine Research Center, National Institute of Allergy and Infectious Diseases, National Institutes of Health, Bethesda, Maryland, United States of America; Emory University, UNITED STATES

## Abstract

The nonhuman primate (NHP) model is often the best experimental model for testing interventions designed to block infection by human pathogens, such as HIV, tuberculosis, and malaria. A physiological model may require the use of a limiting dose of the infectious agent, where only a fraction of animals become infected upon any given challenge. Determining the challenge dose of the pathogen in such experiments is critical to the success of the experiment: using too-high or too-low a challenge dose may lead to false negative results and an excessive use of animals. Here I define an optimized protocol for defining the dose of pathogen that infects 50% of the time (AID_50_); other challenge doses, e.g. AID_80_, can be easily calculated from the same data. This protocol minimizes the number of animals, as well as resources and procedures, while providing an estimate of the AID_50_ within 1.5-fold of the true value.

## Author Summary

Exposing nonhuman primates to infectious pathogens (such as tuberculosis, malaria, or the simian equivalent of HIV) is an important model for testing vaccines or other interventions designed to prevent infection or disease. In fact, demonstrating efficacy in animals is often a requirement before clinical testing in humans can be started. A critical variable in such testing is the dose of the pathogen used: this dose should be similar to what humans would encounter. Using too-high a dose may overcome the intervention and mask a successful approach; using too-low a dose may not be relevant. Often, an optimal dose will lead to “successful” infections only a fraction of the times the animal is exposed. A successful intervention experiment therefore needs to use a precisely calibrated dose of the infectious agent; this calibration can only be done by exposing animals to a range of doses and measuring how often they become infected. Here I define the most parsimonious method for performing this calibration: one that uses the least number of animals and procedures. Given the large number of new pathogens being tested in animal models, adoption of such a parsimonious protocol is both economically and ethically warranted, and will thereby enable favorable review of proposed animal use numbers by Institutional Animal Care and Use Committees.

## Introduction

The nonhuman primate (NHP) can be the best (or only) experimental model for studies of human infectious pathogens and immunological interventions. NHPs represent the preferred animal models for testing vaccines and therapeutics for HIV, tuberculosis, ebola, and malaria. However, significant ethical and economic constraints accompany such models: the most prominent includes a mandate to use as few animals as possible to achieve statistically significant and biologically meaningful results.

In many NHP models, it is desirable to use a limiting infectious dose as a challenge—a dose which only infects a fraction of animals at each exposure. Such exposures are generally considered to be more physiological [1], and therefore better mimic the potential clinical impact of vaccination or therapeutic intervention. In addition, low dose repeated challenges provides significantly more statistical power to discern effects of intervention [1–3]. To date, the most-used example of this is repeated mucosal challenge with SIV or SHIV for testing vaccines [4–18], microbicides [4,13,19–21], or peri-exposure prophylaxes [22].

Limiting-dose challenge models require careful titration of the infectious agent: using a dose that is too high (and not physiological) may lead to a false negative effect of the intervention [16], whereas a dose that is too low will lead to insufficient infections within the time frame of the experiment to yield useful data, consequently using animals for no gain as well as wasting resources. Even a 2-fold error in the challenge dose can significantly impact the power of an experiment. The goal of the analyses presented here was to define a titration protocol that minimized animal use and procedures while estimating the AID_50_ to within 1.5-fold of the true value. While much of the discussion is based on NHP (and the SIV model), the protocol is generalizable to any pathogen and any animal model.

A method for estimating infectious doses was published almost 80 years ago [23], Based on this method by Reed and Muench, one would use as many as 48 animals to estimate the AID_50_. A seminal paper by Spouge [24] used a Bayesian framework to define a method to estimate the AID_50_ (and its precision). However, the goal at that time was to define a minimal dose infecting all animals in an experiment (e.g., AID_99_) following intravenous infection (using a purely stochastic model of infection). Here I model infection based in part on Michaelis-Menten kinetics, with the goal of defining a challenge dose that reproducibly infects a fraction of animals; the statistical analysis is similar to a Logit model from Spouge [24,25]. From this, I define a parsimonious protocol to efficiently estimate the AID_50_ for a pathogen; this protocol will, on average, only use 15 animals.

## Results

The principal goal for titrating an infectious agent is to define the outcome response (i.e., probability of infection) as a function of challenge dose. In this regard, a useful quantity is the “50% animal infectious dose” (AID_50_), the dose which results in infection of 50% of animals after a single challenge. To date, many researchers still rely on modifications of the Reed and Muench method [23], which was never optimized to be parsimonious.

The primary ethical consideration for devising NHP protocols is minimizing the number of animals enrolled while still achieving a statistically significant outcome. Secondary considerations include minimizing the time and number of animal procedures required. In titrating a pathogen for experimental use, these criteria must be balanced against the need to accurately estimate the AID_50_. Since the titrated pathogen will be used in multiple, expensive, NHP-intensive experiments, it is paramount that the AID_50_ be determined to within a relatively small error in order to assure success of those experiments. Even a 2-fold error in the AID_50_ can significantly impact the power of a repeated challenge study.

To define the most efficient protocol for determining the AID_50_, several approaches were modeled by simulation (using experimental data as a guide). Varying input parameters to the possible titration protocols combined with large numbers of simulations allowed the definition of a titration method that minimizes animal use while maximizing accuracy of the estimated AID_50_. The optimal proposed protocol was then applied to titration of two virus strains *in vivo*.

Infection is a stochastic process: even if the challenge dose used in a cohort was the exact AID_50_, the number of animals infected will most likely deviate from 50%, following a binomial distribution. For example, if 8 animals were challenged with a pathogen at the AID_50_, there is only a 27% chance that exactly 4 would be infected. As with any measurement that includes statistical random variation, the precision and accuracy of the estimated AID_50_ from a challenge experiment depends on the number of observations: using more animals per challenge will yield a better estimate.

Indeed, the most accurate determination of the AID_50_ will occur when the largest number of animals are challenged with a dose closest to the AID_50_. Therefore, determining the AID_50_ for a challenge stock of pathogen is typically done by iterative exposures of cohorts of one or more animals to a dilution of the stock [26–28], adjusting the dose between each round to try to achieve 50% infection.

**Fig 1:**
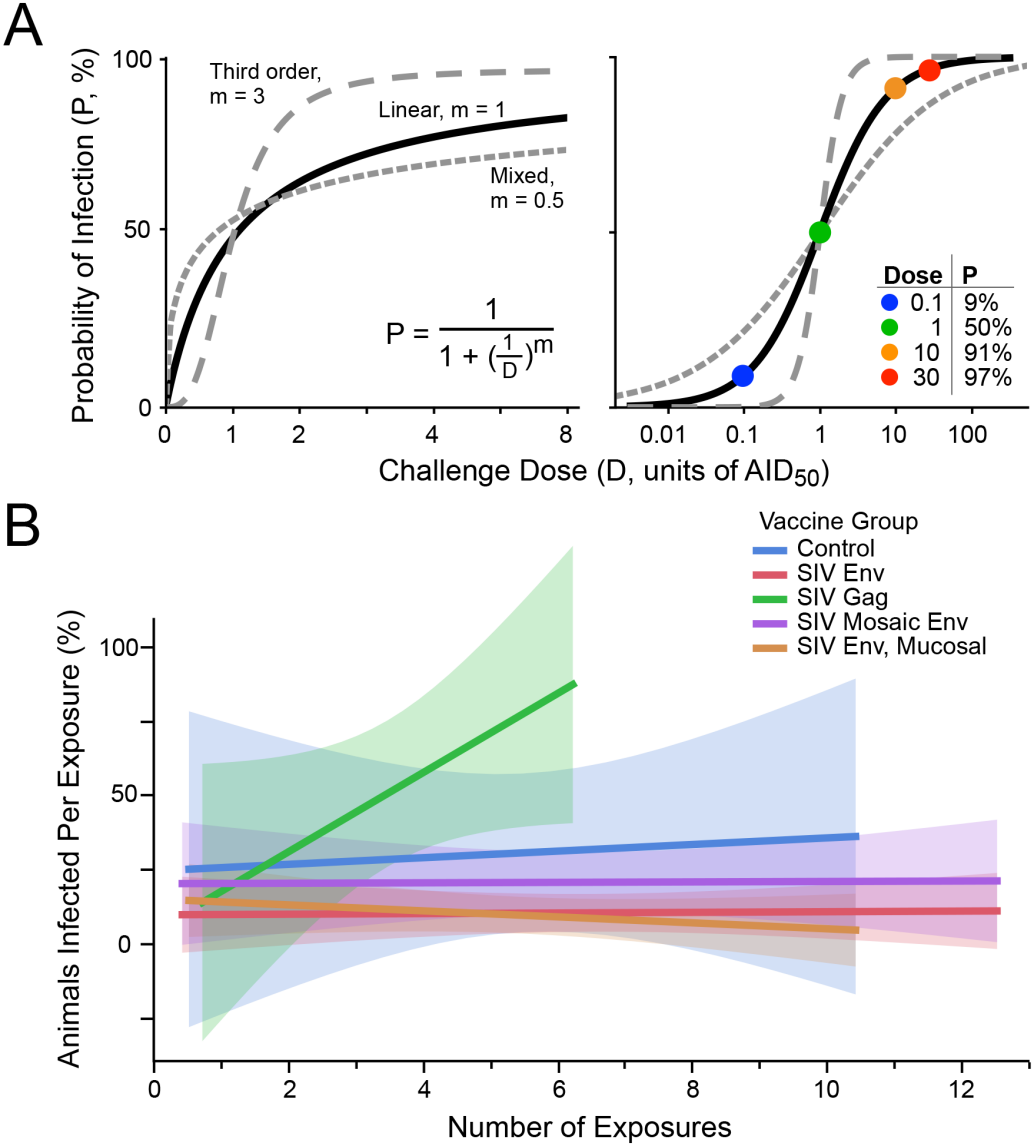
Infection probability as a function of challenge dose. (A) Modeling the probability of infection (P) as a function of the pathogen challenge dose (D). At D = 1, P = 0.5 (i.e., AID_50_, the dose that will infect 50% of the time). The curves for linear kinetics (solid; m = 1), third order kinetics (long dashes; m = 3), and mixed kinetics (short dashes, m = 0.5) are shown on linear (left) and logarithmic (right) scaling. The inset table on the right illustrates some values for P at different doses, for linear kinetics. (B) Five groups of NHP were challenged with a moderate dose of SIVsmE660 (a dose that gave 30% infection in unvaccinated animals, or approximately 0.4 AID_50_). Data are taken from a published study [15]. Env immunized groups were partially protected, showing lower infection rates overall. Shown is the fitted infection rate (linear least squares, with shaded confidence intervals), at each challenge point (i.e., among animals remaining uninfected following prior exposures). There is no decline in infection rate over time, indicating no acquisition of immunity to infection nor selection for innately-resistant animals.

Adjusting the challenge dose between iterative rounds requires an understanding of the relationship between the probability of infection (P) and the challenge dose (D). A precise determination of this relationship is prohibitive; moreover, it may differ by pathogen and experimental conditions. A reasonable hypothesis is that P increases linearly with D at low doses, and asymptotically approaches P = 100% at higher doses. Such a relationship may be modeled by standard first-order linear kinetics, as shown in Fig 1A (where the power term m = 1).

Alternatively, it may be possible that higher order kinetics is suitable, i.e., that infection occurs more likely above a threshold dose, and rapidly saturates above that. This relationship may be modeled by second (m = 2) or third (m = 3; Fig 1A) order kinetics. Furthermore, it is possible that m < 1 is applicable, indicating a mixed reaction (which might occur, for example, by using a swarm of heterogeneous challenge viruses comprising clones with large differences in infection rates, and/or having a distribution in host susceptibility to infection among animals challenged). An example of such a model is also shown in Fig 1A, for m = 0.5.

Most of the analysis in this paper was performed assuming first order kinetics. Indeed, data from an SIV challenge experiment using two doses of virus support the use of first-order kinetics for SIV infection (Table 1). While this relationship may not hold for other pathogens, it is the most reasonable in the absence of data to the contrary. As shown below, the reaction order impacts the efficiency by which AID_50_ is estimated, but does not impact the choice of optimal experimental parameters.

**Table 1:** SIV challenge dose impact on infection rate.

Group	“Low” Dose Challenge^1^		“High” Dose Challenge^1^		Effective Dose Ratio^4^
	Outcome^2^	Effective Dose^3^	Outcome^2^	Effective Dose^3^	
**Unvaccinated**	20/59	0.51 ± 0.13	5/7	2.45 ± 2.14	4.8 ± 4.4
**Vaccinated**	28/256	0.12 ± 0.03	5/21	0.32 ± 0.17	2.7 ± 1.5

Notes:

^1^ “Low” dose challenge: Intrarectal (IR) administration of 1 mL of a 1:5 dilution of an E660 challenge stock. “High” dose challenge: IR administration of 2 mL of undiluted challenge stock: i.e., 5x the concentration, 10x the amount, of the “low” dose.

^2^ Outcome is shown as “number of animals infected” / “total number of exposures”.

^3^ Effective dose is given in units of AID50 (the dose estimated to infect 50% of the cohort after a single administration), and is shown as the estimated value ± standard error based on nonlinear regression to first-order kinetics.

^4^ The ratio of the high to low estimated effective doses assuming first-order kinetics, calculated from the outcome. This ratio should theoretically match the actual infection dose ratio of 5–10. If infection followed higher-order kinetics, the effective dose ratios would be much lower (2.2 and 1.6 for m = 2; 1.7 and 1.4 for m = 3). Thus, first-order kinetics appears to be the best model for SIV infection from these limited data.

The best estimate of the “true” AID_50_ occurs with the most measurements occurring in the linear phase of the response curve (i.e., close to the AID_50_). For example, challenging many animals at a high dose will result in nearly uniform infection, providing little information about the actual dose (was the challenge dose too high by 100x? or 10x?). Similarly, challenging animals at too low a dose, where none or few are infected, also provides little precision for estimating the AID_50_. However, this case is fundamentally different than challenging at too-high a dose: here, most animals remain uninfected, and can be re-challenged with an adjusted (increased) dose. With too-high dose, new animals must be enrolled into the titration study.

To define the optimal titration experiment, an iterative protocol was modeled by Monte Carlo simulation. This iterative approach includes two phases: phase 1, a dose-ranging study, during which the challenge dose is modified following each round, until termination criteria have been met; and phase 2, during which any uninfected exposed animals from phase 1 are repeatedly challenged until infected in order to refine the estimate of the AID_50_.

Re-using exposed but uninfected animals can only be done if exposure does not change subsequent risk of infection. Such a change could occur actively: exposure could “vaccinate” the animal, or engage an innate response, so that the animal becomes more resistant to subsequent challenge. It is also possible that there is a heterogeneity in the innate susceptibility to infection, and over time, repeated exposures will select for such animals. Such a process might skew the statistical analysis of infection data, and one may wish to set a cap on the total number of exposures of any single animal after which data from that animal is censored.

These risks must be evaluated for each pathogen. In the case of SIV, there is no evidence for a decline in infection probability following multiple exposures [29] (Fig 1B). Thus, for this model, uninfected exposed animals can be re-challenged to provide more data for the infection-dose response curve. Nonetheless, the impact of limiting the total number of exposures for any given animal in a titration protocol was evaluated.

**Fig 2:**
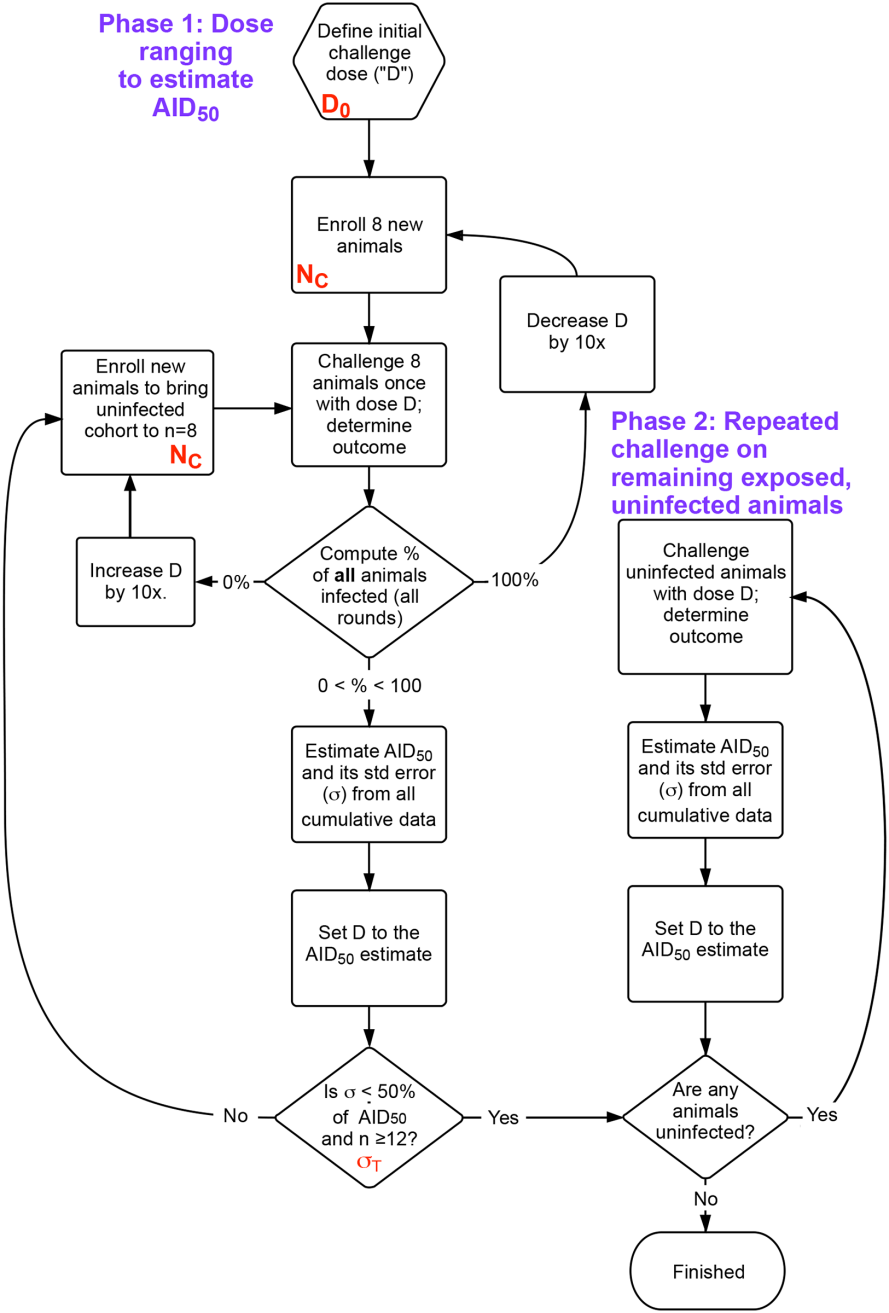
Flow chart of overall procedure. The procedure is divided into two phases. First, an initial dose ranging study that uses the same cohort size (N_C_) at each challenge, where the dose D is refined following an initial guess (D_0_) after each challenge. Once the accuracy of D is within the desired bounds (σ_T_), remaining uninfected animals are moved to phase 2, undergoing challenges until all are infected. The AID_50_ is estimated at each time based on a least-squares regression to the single-parameter equation in Fig 1A, using data from all challenges in phases 1 and 2. n = total number of animals enrolled. Shown in red are the parameters optimized in Fig 3.

The outcome response was defined as in Fig 1A; input parameters to the simulation included the number of animals challenged at each round (N_C_), the starting dose of challenge (initial estimate, D_0_, as a fraction of the AID_50_), the desired precision of the final estimate of AID_50_ (σ_T_), and the maximum number of exposures for any given animal (E_MAX_). The outputs of the simulation were the total number of animals enrolled (n), the total number of rounds during phase 1, and the distribution of estimated AID_50_ for all simulations. The flowchart shown in Fig 2 outlines the protocol.

The accuracy of the estimate of the AID_50_ is greatest when 50% of animals challenged are infected. However, because of the stochastic nature of the infection, there is a good chance that this outcome is possible even with a challenge dose that is not near the AID_50_ (particularly for small values of N_C_). For example, at a 0.25 AID_50_, the probability of infection (P) is 20%; in a cohort of 4 animals using this low dose, the chance of two or more animals becoming infected with one challenge is nearly one in five. Such an outcome would lead to erroneous dose de-escalation, or, if the accuracy criterion was too loose (i.e., a large σ_T_), a termination of the titration with an AID_50_ estimate off by a factor of 4.

In initial simulations, the probability of premature termination (with a final estimated AID_50_ well off the actual value) occurred too frequently when fewer than 12 animals had been enrolled. Hence, the procedure includes a Phase 1 termination criterion that the estimate of AID_50_ must be based on a minimum of 12 animals.

**Fig 3:**
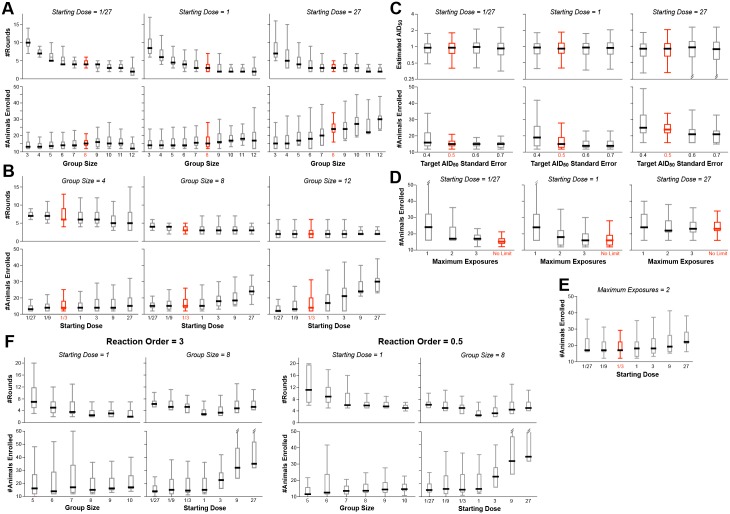
Example results from optimization of procedural parameters. The procedure outlined in Fig 2 was evaluated by Monte Carlo simulation (350–1000 simulations for each set of values). The parameters modeled were: (1) the starting dose (D_0_), ranging from 1/27^th^ to 27x AID_50_; the number of animals challenged at each dose during the dose ranging (group size: N_C_); the target standard error on the estimate of AID_50_ (σ_T_); the maximum number of exposures (E_MAX_) of any single animal; and the order of the kinetics of infection (m). Shown in red are the suggested values of these parameters, i.e., those that resulted in the fewest animals, the fewest rounds, and the most accurate estimate of AID_50_. Where not graphed, parameter values were held constant, as follows: N_C_ = 8, σ_T_ = 0.5, D_0_ = 1/3, m = 1, E_MAX_ = unlimited. (A) The number of animals required is not strongly dependent upon the group size, except in cases where the starting estimate of D_0_ is greater than 1 AID_50_. Thus, in the case where D_0_ is low, the number of rounds required decreases with increasing group size, to a point. (B) The number of animals required increases when D_0_ goes above 1, but the number of rounds (shown for phase 1) is not strongly impacted by D_0_. (C) The number of animals required decreases as the target standard error (σ_T_) on the final estimate of AID_50_ decreases (i.e., more precision requires more data). (D) Decreasing E_MAX_ increases the number of animals required, particularly at low D_0_. If D_0_ is close to 1, then a limit of three exposures performs equally to no limit. (E) When E_MAX_ is limited, then the number of animals required increases when D_0_ is far from AID_50_ in either direction. (F) Example outcomes on parameters when using a infection kinetics with nonlinear order (m = 3 or m = 0.5). The pattern of results is largely similar to first-order kinetics, with similar optima. Bar and whisker plots show the median, interquartile range, and full range excluding outliers.

A large number of simulations were performed, simultaneously varying the four input parameters over reasonable ranges (S1 and S2 Tables). Fig 3 shows the distributions of output values for a selection of the simulations, in order to illustrate how the outcomes depend on these values.

The number of animals required for the study is fairly constant when the initial challenge dose (D_0_) is at or below the actual AID_50_, irrespective of group size (Fig 3A and 3B). This is because of the reuse of uninfected animals for subsequent rounds. In contrast, if the number of exposures per animal (E_MAX_) is limited, the number of animals required increases as D_0_ deviates in either direction from the actual AID_50_ (Fig 3D and 3E).

With small group sizes (N_C_ < 8), the number of rounds in Phase 1 increases dramatically as D_0_ decreases (Fig 3A)—requiring far more time and re-exposures of uninfected animals. This cost in efficiency is not offset by a decreased requirement for total number of animals—hence the recommendation that N_C_ = 8.

If the infectivity of the pathogen is completely unknown, it is may be best to start with a pre-dose ranging study using one or two animals, with a very low dose, and doing step-wise 10-fold increasing challenge doses until infection is achieved: this now can be used as D_0_ for the main protocol.

The major termination criterion for Phase 1 is achieving a standard error on the estimate of AID_50_ (σ_T_) that is below 0.5 (50%). Changing this threshold to higher values reduces the number of animals required (Fig 3C)—but at a higher risk of an inaccurate final AID_50_ estimate. Notably, the risk of underestimating the AID_50_ is greater than overestimating it; if underestimating the AID_50_ is an acceptable risk, then the titration protocol can be made more parsimonious by increasing σ_T_ to 60% or 70%. Doing so does not change the optimal value of 8 for N_C_ (S1 Table).

In models where exposure to the pathogen substantially changes subsequent risk of infection, every animal should only be challenged once irrespective of outcome (E_MAX_ = 1, Fig 3D). Simulations performed with such a restriction showed that the optimal parameters are unchanged, i.e., N_C_ = 8 and σ_T_ = 0.5. Of course, the inability to re-use uninfected animals significantly increases the total number required (Fig 3D and 3E).

Simulations were performed on a model infection system with 3^rd^ order infection kinetics (“threshold infection”; m = 3 in Fig 1A). As shown in Fig 3F, the patterns of outcome dependencies on input parameters is similar to first order kinetics, but requiring more animals and more rounds. This occurs because of the steepness of the response curve (Fig 1A)–i.e., small variations in infection frequency (arising from the stochastic nature of infection) lead to much larger errors in estimating the AID_50_ when the infection frequency is not near 50%.

**Fig 4:**
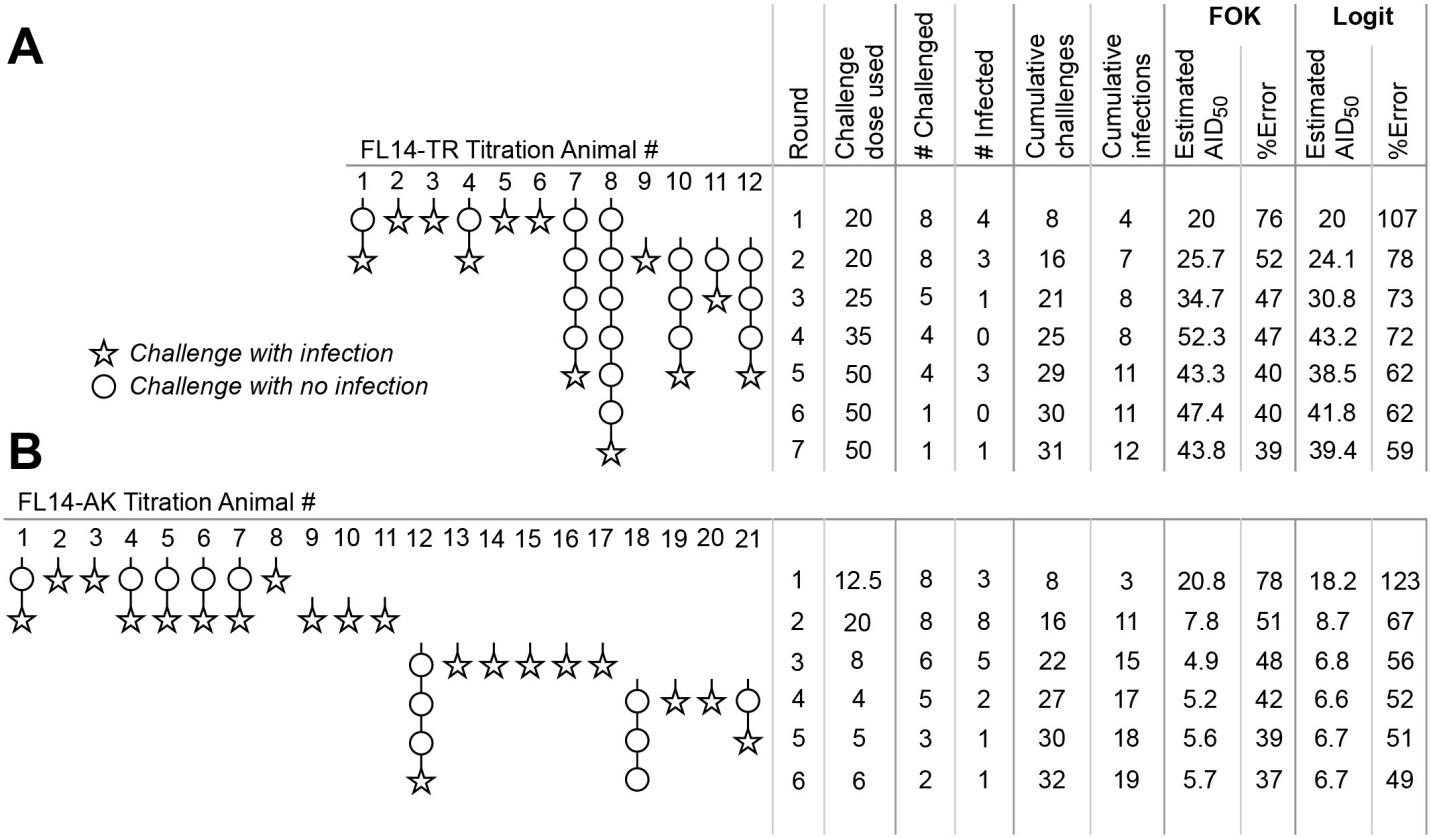
Application of optimized protocol to *in vivo* titrations. The procedure outlined in Fig 2 was used to titrate two different SIV strains *in vivo*. For both strains, challenges and outcomes are represented pictorially, with statistical analyses tabulated to the right. Estimates of AID_50_ and its precision were done using the first order kinetics model described in Fig 1 (“FOK”), or by the Logit model online calculator (“Logit”) [25]. (A) Titration of SIV FL14-TR. (B) Titration of SIV FL14-AK. Note that the protocol was terminated with one animal still uninfected in phase 2; further challenges will not substantively change the estimate of AID_50_.

This protocol was followed to titrate two SIV strains *in vivo* (Fig 4). Titration of FL14-TR (Fig 4A) began with a challenge dose of 20 μl of stock diluted into 1 ml (based on estimates from the TCID_50_ of the stock). 4/8 (50%) of animals were infected, resulting in no change in the estimated AID_50_, which, after one round, had an error of 76%. In round 2 of this challenge dose, only 3 of 8 animals became infected, necessitating an increase in the AID_50_ estimate, to 25 μl. At this point, 12 animals were enrolled and the error on the estimate was 52%, so the decision was made to enter phase 2 (and not enroll additional animals). By the end, with 12 animals, there were a total of 31 challenges with 12 infections at a range of doses, and the final AID_50_ estimate was 50 ± 41%.

Fig 4B illustrates the titration of a SIV FL14-AK stock. Because this stock is more concentrated (by TCID_50_), the initial challenge dose was selected to be 12.5 μl in round 1. 3 of 8 animals were infected, so round 2 used dose of 20 μl—resulting in 100% infection. For round 3, only 6 animals were available, and a much lower dose was used. While the threshold to enter phase 2 was barely met following the 3^rd^ round, a decision was made to enroll four more animals prior to initiating phase 2. The final AID_50_ estimate, based on 21 animals and 32 challenges was 5.7 ± 37%.

Fig 4 also compares statistical estimation of the AID_50_ and its precision based on the first order kinetics model described here, and the Logit model described by Spouge [24,25]; the latter has a web-based calculator. Overall, the progressive estimates of AID_50_ are highly comparable; the confidence interval reported by the Logit model is about 50% higher (presumably, a different interval). Thus, if using the online calculator, multiply the error estimate by 0.67 to convert to the σ_T_ used here, or (equivalently) use a termination σ_T_ that is 1.5x larger than suggested here.

## Discussion

From these data, and based on the optimization criteria (in order of priority: minimum number of animals, minimum number of rounds in Phase 1, maximum accuracy on the AID_50_ estimate), the optimum parameter values were selected. Specifically, the number of animals per cohort (N_C_) should be 8 (with 7 being close to optimal); the target precision (σ_T_) should be 0.5 (with 0.6 being close); and the maximum number of exposures per animal (E_MAX_) should be unlimited (with 3 being close to optimal). If any information about the likely AID_50_ is known (e.g., from previous experiments), then a starting challenge dose (D_0_) should be between 0.1 and 1 AID_50_.

Using as many as 8 animals per round may seem unintuitive—for example, why is N_C_ = 4 less efficient? There are two reasons for this: First, the criterion for precision (σ_T_ < 0.5) is heavily dependent on the total number of exposures in the experiment—thus, using four animals will usually double the number of rounds in the experiment (increasing time and resources) without reducing the eventual number of animals. Second, a four animal cohort presents a much larger risk of a obtaining an outlier outcome (due to the stochastic nature of the infections), leading to incorrect dose adjustment for the following round.

Fig 4B illustrates this risk. In this titration, only 3 of 8 animals were infected despite using what turned out to be a challenge dose that was ~2-fold above the actual AID_50_. Based on Fig 1A, using this dose is expected to infect 68% of animals challenged once; the odds of having 3 or fewer of 8 infected is low (8%) but not insubstantial. Were this the extent of the titration, a future (large) study would have used this or even higher challenge dose. By following the procedure specified here, this statistical anomaly was overcome.

The length of time required to determine the outcome of a challenge will define the length of each round. For example, with a titration of highly pathogenic SIV, infection usually be determinable by day seven and always by day 10. Thus, each subsequent round will likely be initiated two weeks apart. In contrast, during phase 2, this step can be accelerated. For example, an animal might be infected during the first round of phase 2, and then re-challenged one week later when its infection status is still unknown. The animal would become viral positive day 10 (i.e., a few days after the second challenge of round 2), indicating that it actually became infected after the first round and that the subsequent challenge should be discounted. This ambiguity at day seven, fully resolved before day 14 (i.e., 3^rd^ round), does not impact the analysis of the phase 2 data. However, such ambiguity is not appropriate for phase 1, where challenges must be spaced with sufficient delay to positively establish infection.

However, other pathogens may require considerably more time between rounds in phase I. For example, in titrating a stock of SHIV 162p3, we found that some animals did not become viremic until day 14, and adopted a 3 week cycle for titrations. With mTB, infection may not be clinically evident until four weeks post-exposure, and the cycle time needs to be commensurately higher.

The number of animals enrolled in phase 2 is between zero and N_C_ (i.e., the number of uninfected animals remaining from the last round of phase 1). At this point, a fairly accurate estimate of the AID_50_ is available, so phase 2 will usually require no more than 3–4 rounds to infect the remaining animals. Therefore, the total time required for this titration protocol will be, on average, four rounds for phase 1 and three rounds for phase 2. In the example of titration of an SIV, where each round in phase 1 requires two weeks and each round in phase 2 requires one week, the total time for the titration should be expected to be 11 weeks. In our *in vivo* examples (Fig 4), titration of FL14 was finished in 8 weeks, and FL14-AK in 12 weeks. Of course, the total time will depend on the specific requirements of each pathogen and system, and the timing between rounds should be adjusted accordingly.

Many variations of titration protocols were evaluated, including (i) using smaller group sizes during initial rounds of Phase 1 and larger group sizes when narrowing in on the true AID_50_, (ii) other modes of variable group sizes; and (iii) eliminating Phase 2. Most alternatives performed worse in by requiring more animals and/or more time and procedures. The scripts created for the simulations reported here can be modified to test any configuration of the titration protocol that might be considered for a given pathogen.

It should be noted that this protocol returns an estimate of the average AID_50_ for a cohort of animals, and will be valid for an experimental cohort that is matched for relative infection rates. For example, this protocol could be used for titration of a virus used in intravaginal challenge where the infection risk varies by time in the menstrual cycle. The best results will be obtained by challenging both titration and experimental animals at the same point in the menstrual cycle. But as long as the challenge time points are equivalently randomized across the menstrual cycle in both the titration and experimental cohorts, the estimate of the AID_50_ will still be valid.

Most of the modeling assumed a uniform infection rate for the viruses in the challenge stock (i.e., by setting m = 1). If a swarm of genetically distinct viruses is used, in which clonal variation in infection efficiency is present, then the result may be a mixed kinetics of infection (i.e., m < 1). Modeling even a severe example of this (m = 0.5) resulted in the same optimal parameter values, albeit the total number of animals (and rounds) may increase (or even decrease in some cases).

A somewhat different model for the infecting dose response was used by Spouge his seminal paper [24]. While the motivations and goals of that paper were different than for this paper, either model can be used to estimate the AID_50_ (Fig 4). A difference between the Spouge model and the model used here is in the estimate of the precision of the AID_50_ (σ_T_), which differ by a factor of about 1.5. Consequently, if using the convenient online calculator (select the “Logit” fit) [25], multiply the error estimate by 0.67 to approximate σ_T_ as used here.

In some experiments, it may be desirable to use a higher (e.g., AID_80_) or lower (e.g. AID_20_) challenge dose. These doses can be estimated from the following formula (assuming, based on the estimated AID_50_, where “x” is the desired percentage of successful infections:

$$AID_{x}=\;\frac{AID_{50}}{\left(\frac{100}{x}\right)-1}$$

In summary, an optimized pathogen titration protocol is presented (Fig 2) to determine with good precision the limiting infectious dose of a pathogen, a dose which may be critical for testing interventions [16]. On average (90% range in parentheses), the protocol will enroll 15 (12–25) animals, require 3 (2–5) phase 1 rounds, and result in an estimate of the AID_50_ within 40% of the true value.

The protocol defined here is generalizable to any pathogen and any animal model. However, it is most critical for NHP models, where ethical considerations mandate minimization of animal use. This protocol and the analyses reported here will aid in the preparation of an Animal Study Proposal (ASP) for the purposes of defining the characteristics of an NHP-infectious agent. Finally, these analyses provide a statistical justification for animal numbers in the ASP, as required for Institutional Animal Care and Use Committee (IACUC) review and approval. Importantly, by following the method described here, investigators can minimize the number of animals and resources needed to define an infectious pathogen challenge dose in NHPs.

## Methods

Data in Fig 1B and Table 1 from a large vaccine/challenge study, VRC 10–332, the results of which have been published [15]. Data in Fig 4 represent titration of two VRC-amplified challenge stock of SIV strains, administered intrarectally, in rhesus macaques (VRC 13–444). Blood was collected weekly during the challenge phase for viral load quantification.

All simulations and statistical analyses were performed in JMP versions 10 and 11 (SAS Institute, Cary, NC). Simulation scripts and data tables are available by request to the author. A simple JMP document that includes a script to calculate the AID_50_ as well as the precision (σ_T_) is also available by request. In this context, σ_T_ is the approximate standard error of the regression; according to the software documentation, “it is formed by the product of the RMSE and the square root of the diagonals of the derivative cross-products matrix inverse.”

### Ethics Statement

All in vivo procedures were carried out in accordance to institutional, local, state, and national guidelines and laws governing research in animals including the Animal Welfare Act. The animal protocols “VRC ASP 10–332” and “VRC ASP 113–444” and the procedures were reviewed and approved by the Animal Care and Use Committee (ACUC) of both the Vaccine Research Center (in accordance to all the NIH policy and guidelines) as well as the Institutional Animal Care and Use Committee (IACUC) of Bioqual, Inc. where non-human primates were housed for the duration of the study. Bioqual Inc., and the NIH are both accredited by the Association for Assessment and Accreditation of Laboratory Animal Care (AAALAC) and are in full compliance with the Animal Welfare Act and Public Health Service Policy on Humane Care and Use of Laboratory Animals. In accordance to the institutional policies of both institutions, all compatible non-human primates are always pair-housed, and single housing is only permissible when scientifically justified or for veterinary medical reasons, and for the shortest duration possible.

Non-human primates were housed in appropriately sized caging according to the *Guide for the Care and Use of Laboratory Animals*, *8th ed*., and supplemented with a variety of enrichment toys, treats, fresh produce, and foraging devices. Water was offered *ad libitum* and monkeys were fed primate biscuits (Monkey Diet, 5038, Lab diet, St. Louis, MO) twice daily. Animal holding rooms were maintained on a 12 hour light/dark cycle, room temperature of 60-70F, and relative humidity between 30 to 70% as standard practice.

The studies being performed support HIV/AIDS research and, as addressed in the Weatherall report, non-human primates were an appropriate animal model for this infectious disease. In order to ensure that animal use was not duplicated, literature searches were performed using several key words related to the study. Specific intervention and endpoint criteria tables were integrated into the protocols. Facility veterinarians were familiarized with the disease model and progression to minimize any associated pain or distress, and to perform euthanasia as directed in the approved ACUC and IACUC protocols.

## Supporting Information

S1 Table Tabulated outcome of simulations testing algorithmic parameters. All combinations of group size (n = 3 to 12 by increments of 1), starting challenge dose (D = 0.037 to 30 by factors of 3), and target AID_50_ precision (σ_T_ from 0.4 to 0.7 by increments of 0.1) were simulated with 350 iterations. The mean and standard deviation of the outcomes is shown for each set of inputs. Note that the ideal estimated AID_50_ would be 1.0, and the ideal Log_10_(AID_50_) is 0.0. In these simulations, there is no limit to the number of exposures that would be given to any single animal.
(XLSX)

S2 Table Impact of limited exposures on experimental needs. 1000 simulations were performed across a range of starting challenge dose (D = 0.037 to 30 by factors of 3), and maximum number of exposures for any given animal (1, 2, 3, or unlimited; the latter are the same as in S1 Table), while holding the group size (n = 8) and target AID_50_ precision (σ_T_ = 0.5) fixed. Outputs are the same as in S1 Table.
(XLSX)
